# Epoxy Clerodane Diterpene Attenuates the Differentiated Adipocyte Hypertrophy and Enhances Mitochondrial Metabolism

**DOI:** 10.1002/open.70230

**Published:** 2026-06-18

**Authors:** Sahar Abdulaziz AlSedairy, Pandurangan Subash‐Babu, Maha H. Alhussain, Manal Abdulaziz Binobead, Ali A. Alshatwi

**Affiliations:** ^1^ Department of Food Science and Nutrition College of Food and Agricultural Sciences King Saud University Riyadh Saudi Arabia

**Keywords:** adipocyte hypertrophy, adipogenesis, epoxy clerodane diterpene, inflammatory mediators, metabolic dysfunction, mitochondrial activity

## Abstract

Adipocyte hypertrophy is an obesity‐related metabolic dysfunction, which is frequently associated with decreased mitochondrial activity during adipocyte development. The current study aimed to assess the potential of epoxy clerodane diterpene (ECD) (IUPAC: 5R, 10R)‐4R, 8R‐dihydroxy‐2S, 3R:15, 16‐diepoxycleroda‐13(16), 17, 12S:18,1S‐dilactone) extracted from *Cassia tora* on adipocyte differentiation, lipid accumulation, mitochondrial function, and inflammation. Human bone marrow mesenchymal stem cells (hMSCs) were stimulated into adipocytes using the standard differentiation medium. The methodological design included the evaluation of ECD cytotoxicity, lipid accumulation, mitochondrial membrane potential, qRT‐PCR, and ELISA. ECD didn’t significantly reduce the cellular viability; however, it decreased lipid accumulation by 65%, 87%, and 87.5% at doses of 2, 4, and 8 μM, respectively. Also, at 4 µM of ECD, it decreased adipocyte hypertrophy, increased mitochondrial membrane potential, raised the expression of thermogenesis‐related genes (UCP‐1, PPARγC1α, SREBP1c), decreased the expression of adipogenic proteins (C/EBPα, PPARγ), increased adiponectin levels, and reduced inflammatory markers (IL‐4, TNF‐α) compared to untreated controls. Thus, ECD may hold tremendous promise as a natural agent for controlling adipogenesis, and its impacts on lipid metabolism, mitochondrial function, and inflammatory responses demonstrate its potential for therapeutic use in the treatment of obesity and associated metabolic diseases.

## Graphical abstract

Epoxy Clerodane Diterpene (ECD) significantly reduces adipocyte hypertrophy and lipid accumulation in human mesenchymal stem cell (hMSC)‐derived adipocytes. ECD improves mitochondrial membrane potential (ΔΨm), increases thermogenic regulators (UCP‐1, PPARγC1α), decreases adipogenic markers (C/EBPα, PPARγ), and reduces inflammatory mediators (TNF‐α, NF‐κB), exceeding orlistat in preventing adipogenesis.

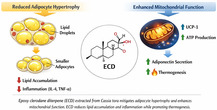

## 1 Introduction

The process of adipocyte differentiation, also known as ‘adipogenesis,’ is a complex biological process in which preadipocytes differentiate into fully developed adipocytes, which are responsible for lipid storage and the regulation of energy balance [1]. While the process plays a critical role in ensuring metabolic homeostasis, its dysregulation can lead to abnormal fat deposition, which manifests clinically as obesity, hyperlipidemia, and adipocyte hypertrophy—afflictions associated with an increased susceptibility to metabolic disorders, including type 2 diabetes and cardiovascular disease [2].

Adipogenesis is modulated by hormonal signals, transcription factors, nutritional status, and environmental cues. The PPARγ and C/EBPα transcription factors regulate genes responsible for lipid absorption and metabolism, which in turn promote adipocyte growth [3]. Insulin and glucocorticoids are required for increased adipogenesis, although inflammation and oxidative stress might hinder their function [4]. Furthermore, dietary components, particularly high‐fat meals, may exacerbate adipocyte hypertrophy and increase lipid storage, compromising metabolic health [5].

Several treatment strategies are being researched for hyperlipidemia and hypertrophy. Pharmacological interventions, such as fibrates and statins, aim to lower cholesterol levels and improve metabolic profiles, while lifestyle changes, including diet and exercise, are essential for promoting healthy adipocyte function [6]. Furthermore, innovative therapy techniques, such as natural chemicals and bioactive compounds, have shown promise in modulating adipogenesis and reducing lipid accumulation in adipocytes [7]. Understanding the complicated mechanisms that drive adipocyte differentiation, as well as the factors that influence adipogenesis, is crucial for developing effective treatments for hyperlipidemia and obesity‐related comorbidities.

Epoxy Clerodane Diterpene (ECD) is a naturally occurring chemical obtained mostly from plant sources, particularly *Caryophyllaceae* species like *Daphne* and *Plectranthus.* This chemical has received attention in recent years due to its wide range of biological activities, including anti‐inflammatory, anti‐cancer, and anti‐obesity properties [8, 9, 10]. ECD’s distinct chemical structure, featuring a bridging bicyclic framework, improves its capacity to modulate various cellular signaling pathways, positioning it as a potentially viable therapeutic option [11].

An especially promising research area for ECD is its potential application in combating obesity, a rising global health concern associated with numerous metabolic disorders, including type 2 diabetes, cardiovascular diseases, and specific cancers. These systems are important for treating obesity because they regulate fat accumulation and energy expenditure. The novelty to accompany the utilization of ECD as a method of inhibiting adipogenesis and obesity is derived from the varied mechanisms of action, which are otherwise absent in current anti‐obesity treatment. This has the potential to facilitate the creation of new drugs that not only induce weight loss but also improve overall metabolic health, thereby resulting in significant progress in treatment strategies employed for obesity. Furthermore, the new chemical topology of ECD will also permit the design of new derivatives with improved potency and selectivity, which will open up new pathways for research studies and clinical applications in metabolic diseases.

So, the purpose of the current study was to thoroughly assess the biological activity of ECD isolated from the seeds of *Cassia tora* in terms of adipogenesis and metabolic function. The study aimed to determine its effects on cell viability, lipid accumulation, mitochondrial membrane potential, gene expression of thermogenic and adipogenicity markers, and inflammatory mediators in human mesenchymal stem cell‐derived adipocytes.

## 2 Results and Discussion

**FIGURE 1:**
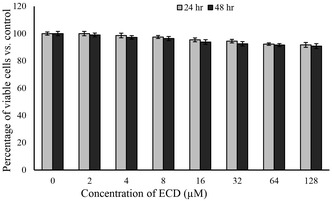
In vitro cytotoxic effect of ECD on preadipocytes. The cellular viability was assessed by MTT assay at 24‐ and 48‐hours post‐treatment with different concentrations of ECD. All data were statistically significant compared to the control.

In the current study, analysis of the impact of ECD on cell viability in preadipocytes showed that even at higher ECD doses, minimal cytotoxic effects were achieved, thereby affirming its potential safety for therapeutic applications. The viability of ECD‐treated preadipocytes wasn’t significantly affected at all concentrations. Cell viability decreased slightly at higher concentrations (64 and 128 µM), but not significantly compared to the negative control and below IC_10_ criteria. At the highest concentration (128 µM) of ECD, there was only an 8.3% and 9.2% reduction of cell viability in 24 and 48 h settings (Figure 1). Particularly, even at 128 µM concentration, ECD caused only an 8.3% reduction in cell viability after 24 h and a 9.2% reduction in cell viability after 48 h, both of which were below the IC_10_, IC_25_, and IC_50_ levels. These findings indicate that ECD is not responsible for evoking significant cytotoxic activity, making its applications suitable for requiring minimal cellular injury. This finding agrees with observations from numerous studies evaluating the safety regimes of diterpenoids and related compounds, which are often screened for cytotoxic activity in preclinical trials of anti‐inflammatory, anticancer, and other therapeutic applications [9]. In general, the coexistence of low cytotoxicity and bioactivity is beneficial, especially for agents to be developed for the treatment of chronic diseases, where the survival of the cell is critical [12]. Furthermore, the outcome of an acute toxicity study revealed that the intraperitoneal and subcutaneous administration of ECD in incremental doses up to 320 mg/kg body weight did not cause any behavioral abnormalities or lethality in indomethacin‐induced gastric ulcers in rats [13]. The findings of this research reflect that ECD, with its attribute of minimal cytotoxicity, is a potential agent for applications requiring the least cellular damage. Findings from the current research conform to a broad body of literature reporting the safe use of diterpenes in medical applications, as they provide a biological and relatively less harmful alternative to manmade compounds [13, 14, 15, 16].

**FIGURE 2:**
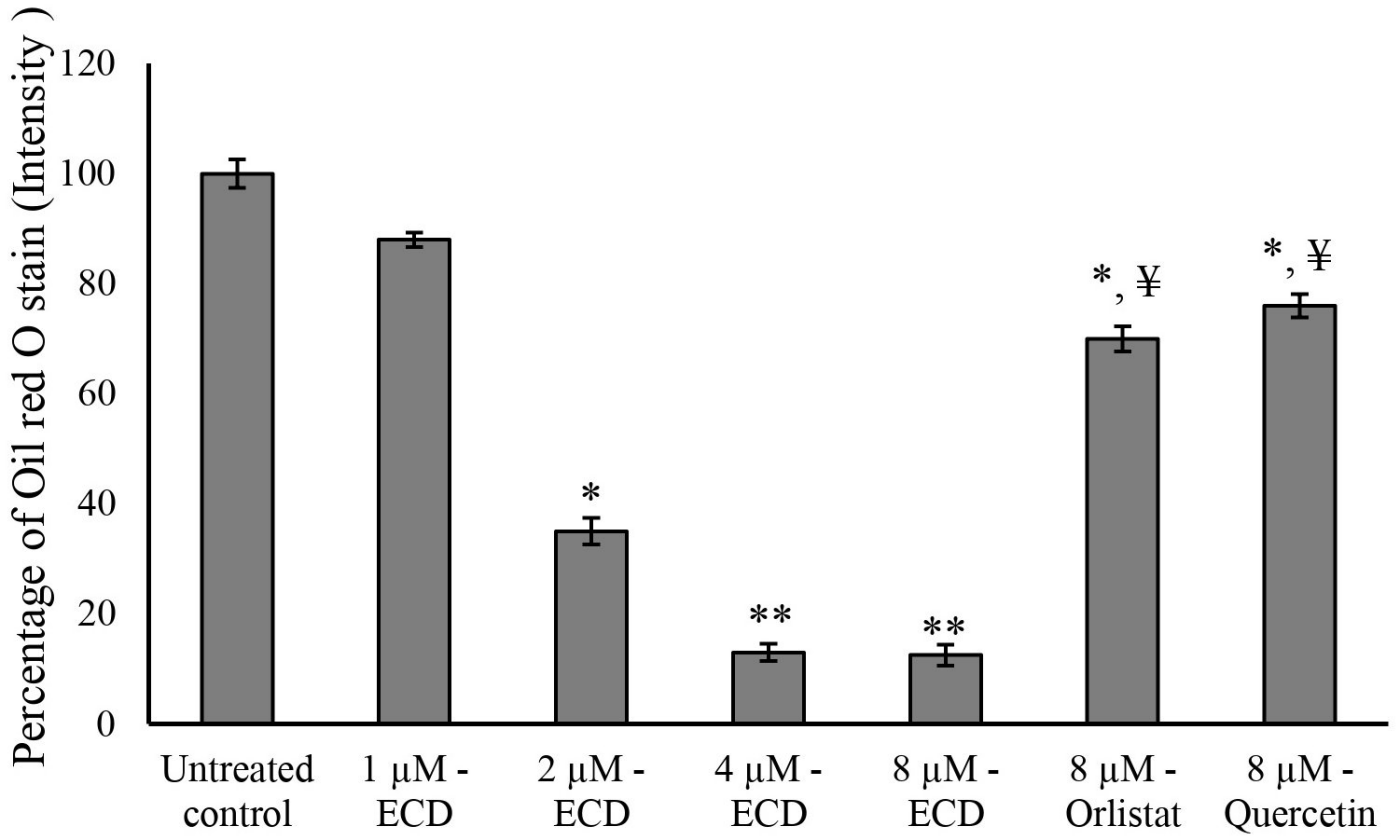
Assessment of lipid accumulation by Oil red ‘O stain. Quantification of Oil red ‘O stain accumulation in preadipocytes following the ECD, Orlistat, or Quercetin treatment for 14 days. Data were expressed as means ± SD. * and ** Statistically significant by comparison with untreated control at *p *≤ 0.05 and *p *≤ 0.001, respectively. ^¥^ Statistically significant by comparison with 8 µM ECD‐treated cells (*p *≤ 0.001).

The study of the ECD‐induced inhibition of adipocyte maturation revealed a significant reduction in lipid accumulation, especially with higher dosages. Based on the cell viability assay results, 1, 2, 4, and 8 μM doses of ECD were chosen to determine the lipid accumulation inhibitory potential. ECD at doses of 4 and 8 μM effectively controlled adipocyte lipid accumulation, as confirmed by oil red O quantification (Figure 2). At doses of 2, 4, and 8 μM of ECD, the lipid levels were reduced by approximately 65%, 87%, and 87.5%, compared to untreated cells. The lipid accumulation inhibition effect of ECD was compared with orlistat and quercetin (8 μM each) for 14 days. Orlistat and quercetin induced 30% and 24% reduction of lipid accumulation, respectively, compared to ECD (85%) (Figure 2). ECD at dosages of 4 and 8 μM significantly curbed lipid accumulation. Quantitative analysis with Oil Red staining also confirmed a reduction in lipid content by nearly 65%, 87%, and 87.5% with dosages of 2, 4, and 8 μM, respectively. Furthermore, in comparison with proven inhibitors of lipid accumulation like orlistat and quercetin, ECD was more effective. Conversely, the control drugs, orlistat and quercetin, given at the same dosage of 8 μM, caused lipid accumulation reductions of 30% and 24%, respectively, while ECD achieved an 85% reduction of lipid accumulation in 14 days of treatment. This significant variance points to the potential of ECD as a more effective alternative to current adipogenesis inhibitors. ECD’s significant inhibition of lipid accumulation is in line with other studies of the anti‐adipogenic activity of natural compounds, specifically diterpenoids. Diterpenoids, such as ECD, modulate key signaling pathways involved in adipocyte differentiation, namely the peroxisome proliferator‐activated receptor gamma (PPAR‐γ), the CCAAT/enhancer‐binding proteins (C/EBP), and the glucose transporter type 4 (Glut4) pathway, while also suppressing the synthesis of cell cycle proteins that act as key regulators of lipid metabolism [17, 18]. Earlier research has also proven that natural compounds like quercetin can block adipogenesis by interfering with these molecular mechanisms [19]. The greater efficacy of ECD in this study may, however, indicate new or more potent mechanisms of action, so further research on its specific targets is needed.

**FIGURE 3:**
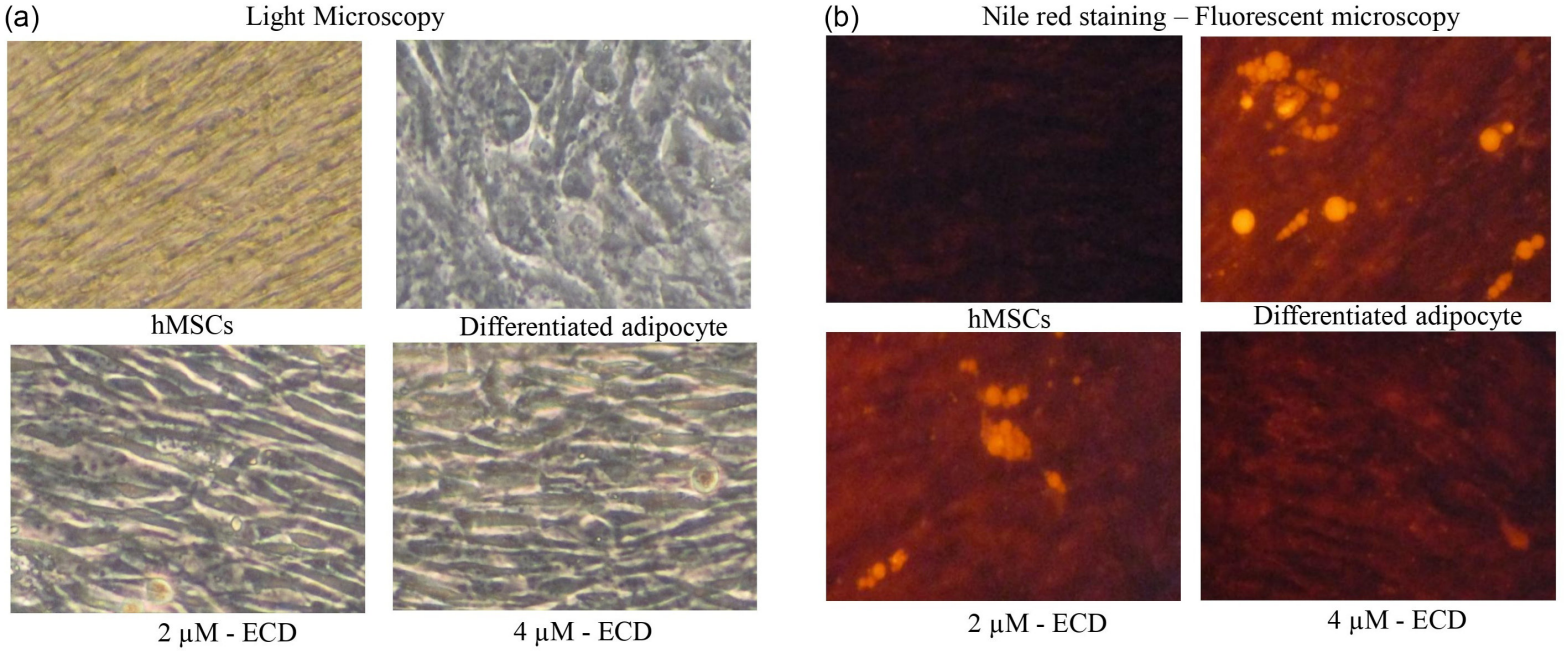
Assessment of lipid accumulation by Nile red staining. Determination, differentiation, and intracellular lipid levels using Nile red staining in ECD‐treated preadipocytes after 14 days. Adipocytes were treated with ECD at 2 µM and 4 µM concentrations. (a) Light microscopy images, (b) Fluorescent microscopy images.

The treatment with ECD significantly reduced both lipid accumulation and adipocyte hypertrophy compared to the control group. Treatment with ECD at 2 and 4 µM concentrations caused a notable decrease in lipid accumulation and adipocyte hypertrophy, with the 4 µM concentration showing a greater effect compared to the 2 µM concentration. Nile red analysis confirmed that vehicle control showed hypertrophic and increased cell size (Figure 3a) and red fluorescence in adipocytes after 14 days (Figure 3b). The light microscopy images showed that the untreated cells were hypertrophic adipocytes with circular cell morphology. Meanwhile, treatment with ECD reversed the circular cell shape to a linear shape in 2 and 4 µM doses. Nile red analysis revealed that ECD treatment significantly decreased lipid accumulation and adipocyte hypertrophy when compared to the untreated control. Most interestingly, the 4 µM dose of ECD reduced adipocyte hypertrophy and lowered lipid accumulation more than the 2 µM dose. Hypertrophic adipocytes are linked to metabolic imbalance, insulin resistance, and inflammation during obesity, which is a result of an abnormal accumulation of lipids in adipocytes, causing them to become larger and resulting in the malfunctioning of adipose tissue [20]. This information supports the fact that ECD is a promising therapeutic compound for controlling adipose tissue growth and improving metabolic health. These findings are also consistent with earlier studies showing that clerodane diterpene can suppress adipocyte hypertrophy and activate lipid metabolism by modulating the pathways involved in lipid storage and differentiation [17]. The capacity of ECD to suppress hypertrophy at very low concentrations further indicates its promise for the treatment of obesity‐related disorders, especially those resulting from excessive lipid accumulation in adipocytes.

**FIGURE 4:**
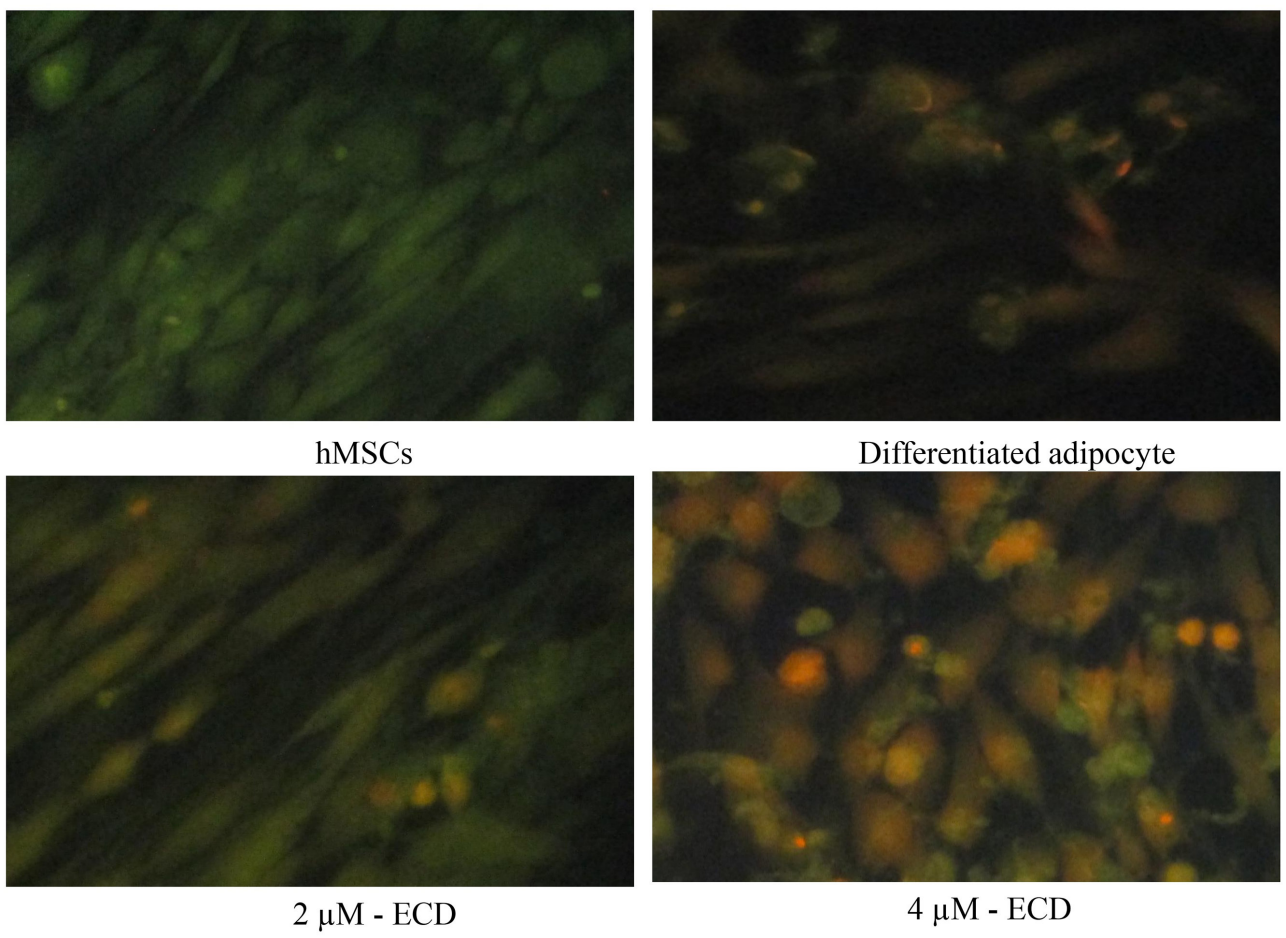
Determination of mitochondrial membrane potential using JC‐1 staining. hMSCs were treated with 4 µM of ECD for 14 days. The qualitative assessment of Jc‐1 staining was achieved by fluorescent microscopy.

Mitochondrial dysfunction, often reflected by the decrease in membrane potential, has been identified as a key cause of metabolic diseases like obesity and resistance to insulin [21]. JC‐1 analysis revealed the vehicle control adipocytes with green fluorescence after 14 days (Figure 4). ECD‐treatment significantly increased the mitochondrial transmembrane potential, which might indicate the increased biogenesis and active shuttle transport at 2 and 4 µM doses. Most interestingly, at the 4 µM dose, ECD induced higher mitochondrial red fluorescence than at the 2 µM dose. Treatment with ECD at 2 and 4 µM significantly restored mitochondrial membrane potential, as reflected through the change from green to red of JC‐1 staining, reflecting the improvement in mitochondrial function. Re‐established mitochondrial membrane potential is possibly mediated by increased mitochondrial biogenesis as well as elevated metabolite transport, both of which are critical in maintaining cellular energy homeostasis. A previous study established that the alcoholic extract of the plant *Tinospora cordif*olia, which is rich in ECD, significantly reduced the level of markers of inflammation (NF‐κB) as well as senescence (HSP70) and prevented glutamate‐induced mitochondrial dysfunction in Wistar rats via primary cerebellar neuronal cell cultures [22]. The ability of ECD to correct or augment mitochondrial membrane potential, therefore, identifies its potential as a therapeutic option to propagate mitochondrial well‐being and efficiency in adipocytes.

Adipocytes treated with 4 µM of ECD showed significant reductions in triglycerides, free glycerol, and HDL levels, indicating a significant lipid‐lowering activity. Treatment with ECD at 4 µM reduced triglycerides more effectively than orlistat or 2 µM. The levels of triglyceride, free glycerol, and glyceraldehyde phosphate dehydrogenase (GAPDh) were significantly decreased in a 4 µM dose of ECD‐treated maturing adipocytes (*p* ≤ 0.01) (Table 1). The lipid‐lowering effect was significantly better than that of orlistat. In addition, a significant decrease in triglyceride levels was observed at a 4 µM dose of ECD treatment, compared to untreated adipocytes. Also, it was higher than its levels in cells treated with quercetin, orlistat, or the lower dose of ECD (2 µM). This capacity to lower cholesterol is critical in treating metabolic diseases such as obesity, as elevated triglycerides and poor lipid metabolism increase the risk of cardiovascular disease [6]. The increased efficacy of ECD compared to orlistat demonstrates the superior benefits of employing natural compounds to treat lipid diseases while avoiding the adverse effects often associated with manufactured medicines. These findings are consistent with previous research on natural products, such as clerodane diterpenoids isolated from *Dodonaea viscosa*, which were found to modulate lipid metabolism by inhibiting ATP citrate lyase, an enzyme involved in hyperlipidemia and hypercholesterolemia [23]. Another study found that clerodane diterpenes derived from *Casearia grewiifolia* can downregulate proprotein convertase subtilisin/kexin type 9 (PCSK9), an enzyme associated with hypercholesterolemia, via inhibiting LDLR recycling [24].

**TABLE 1:** Effect of ECD on triglyceride, free glycerol levels, and GAPDH activity in matured adipocytes after 14 days.

Groups	Triglyceride, mg/dl	Free glycerol, mg/dl	GAPDH Activity^a^
Untreated adipocyte	5.28 ± 0.31	4.27 ± 0.24	0.16 ± 0.06
2 µM of ECD‐treated adipocyte	3.68 ± 0.22*	3.11 ± 0.37*	0.11 ± 0.03*
4 µM of ECD‐treated adipocyte	1.72 ± 0.16**	2.34 ± 0.16**	0.04 ± 0.02**
4 µM Quercetin‐treated adipocyte	3.93 ± 0.41*	3.69 ± 0.47*	0.12 ± 0.04*
4 µM Orlistat‐treated adipocyte	4.35 ± 0.28	3.81 ± 0.35	0.11 ± 0.02

*Note:* Data are expressed as means ± SD (*n* = 6).

a 1 unit = 1 nmol/mg protein.

*: *p* < 0.05.

**: *p* < 0.01 compared with untreated control.

**FIGURE 5:**
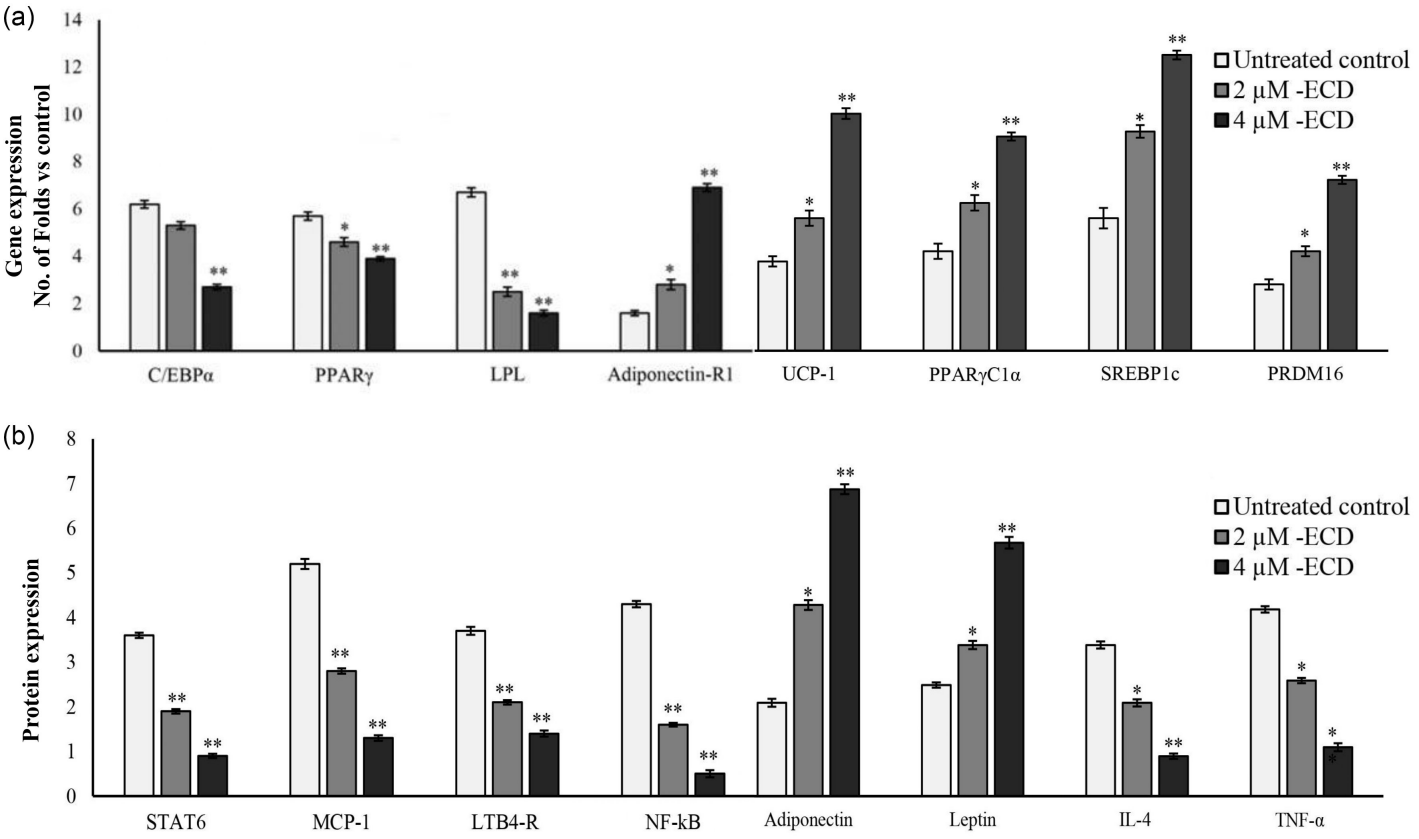
Evaluation of the adipocyte metabolism and mitochondrial biogenesis. The effect of ECD on maturing adipocytes was assessed 14 days post‐treatment by (a) qRT‐PCR and (b) ELISA. Data were expressed as means ± SD. * and ** Statistically significant comparison with the untreated control at *p *≤ 0.05 and *p *≤ 0.001, respectively.

The study discovered that ECD dramatically boosted the expression of key genes involved in the adipocyte mitochondrial function and thermogenesis, including UCP‐1, PPARγC1α, SREBP1c, and PRDM16. These genes are significant regulators of energy expenditure, with uncoupling protein 1 (UCP‐1) playing a critical role in thermogenesis, which boosts heat generation in brown and beige adipose tissue [25]. Based on the above biochemical parameters and lipid accumulation analysis, the ECD doses of 2 and 4 µM were chosen as the most effective. qRT‐PCR analysis revealed the upregulation of the genes related to adipocyte mitochondrial efficiency (UCP‐1, PPARγC_1_α, SREBP1c, and PRDM16) (Figure 5a). Also, the expression levels of mitochondrial thermogenesis‐associated genes increased compared to untreated cells. In the present study, 4 µM ECD treatment significantly decreased the expression levels of C/EBPα, PPARγ, and LPL, and significantly increased the levels of Adiponectin‐R1, when compared to untreated maturing adipocytes (*p* < 0.01) (Figure 5a). The increased expression of these mitochondrial‐associated genes in ECD‐treated adipocytes increased the mitochondrial biogenesis and thermogenic activity, both of which are essential for improving metabolic efficiency and curing obesity‐related metabolic disorders. ECD treatment significantly decreased the expression of C/EBPα, PPARγ, and LPL. These genes regulate adipocyte growth and triglyceride absorption [26, 27]. Interestingly, ECD greatly increased the expression of adiponectin‐R1, an adiponectin receptor that enhances insulin sensitivity and has anti‐inflammatory properties. Increased adiponectin‐R1 expression in ECD‐treated adipocytes suggests that ECD may improve metabolic health by enhancing adiponectin signaling, which is typically disrupted in obese patients [28]. *Polyalthia longifolia*’s clerodane diterpene suppressed MDI‐induced Akt/mTOR activation and cell cycle protein expression during adipogenesis, supporting previous findings [17]. Overall, the results indicated that ECD had a mixed effect on adipocyte gene expression, enhancing mitochondrial efficiency and thermogenesis while lowering adipogenesis and improving insulin sensitivity. The changes in gene expression suggest that ECD can improve metabolic function and prevent or treat obesity‐related illnesses by targeting both adipocyte development and energy expenditure pathways.

Quantification of protein levels in ECD‐treated adipocyte secreted microparticles showed higher levels of adiponectin and leptin, which are associated with energy sensors and mitochondrial functional regulators [18]. Meanwhile, lower levels of insulin resistance‐related proteins, such as IL‐4 and TNF‐α, were observed (Figure 5b). Most notably, insulin resistance and obesity‐associated immune disorders, such as progressive protein levels, IL‐4, and TNF‐α levels, have been suppressed by ECD treatment. In addition, an increased adiponectin and decreased leptin level have been directly proportional to the increased adipocytes’ fatty acid oxidation and mitochondrial metabolic oxidation (Figure 5b). The metabolic inflammation‐associated signaling proteins, such as STAT6 (a transcription Factor that mediates direct repression of inflammatory enhancers and limits activation of alternatively polarized macrophages) [11], MCP‐1, NF‐kB, and LTB4‐R expressions have been decreased in ECD‐treated adipocytes when compared to untreated maturing adipocytes (Figure 5b). ECD raised the expression levels of adiponectin and leptin, two well‐known energy sensors and mitochondrial function regulators, resulting in higher fatty acid oxidation, improved insulin sensitivity, and decreased lipid storage in adipocytes [29]. ECD reduced insulin resistance‐associated proteins such as IL‐4 and TNF‐α, which are connected to obesity‐related immunological dysfunction and insulin resistance [30]. ECD lowered metabolic inflammation‐related signaling proteins, including STAT6, MCP‐1, NF‐κB, and LTB4‐R. STAT6 suppresses inflammatory enhancers and limits macrophage activation, particularly those that lead to a pro‐inflammatory state in adipose tissue [31]. MCP‐1, a chemokine, draws macrophages to fat tissue, causing inflammation. NF‐κB and LTB4‐R are key regulators of inflammation and metabolic dysfunction [32]. These findings suggest that ECD could reduce chronic low‐grade inflammation, a significant source of metabolic issues in obesity, while simultaneously enhancing metabolic health and mitochondrial efficiency. Following these findings, Antonisamy et al*.* (2014) found that ECD reversed indomethacin‐induced increases in ulcer index (UI) and MPO activity, while also downregulating PGE2 and reducing IL‐4 and IL‐10 [8]. Zheng et al*.* (2019) found that a clerodane diterpene inhibited NF‐κB in an AOM/DSS‐induced mouse model [33].

## 3 Conclusion

According to the current study, ECD significantly improved inflammation, lipid metabolism, and adipocyte function. ECD improved fatty acid oxidation, decreased lipid formation in immature adipocytes, and boosted mitochondrial efficiency. Additionally, it inhibited important pro‐inflammatory indicators and signaling pathways linked to metabolic inflammation and insulin resistance. ECD’s abilities to modulate both metabolic and inflammatory processes make it a promising therapeutic agent for the management of obesity‐related diseases like metabolic syndrome and insulin resistance. The result of this study provides an understanding of the potential of ECD as a multifunctional therapeutic agent. ECD’s ability to increase mitochondrial activity, inhibit adipogenesis, and reduce inflammation in adipocytes is a new combination of activities for a natural compound, and it indicates that it has the potential to target both metabolic and inflammatory pathways at the same time. Besides, ECD is superior to traditional drugs such as orlistat in suppressing fat accumulation and inflammatory proteins, making it a potential candidate for the treatment of obesity and insulin resistance. Further studies should build on these novel findings by exploring ECDs’ in vivo action, long‐term safety, and molecular mechanisms, which may broaden their therapeutic applications in adipocyte metabolic disease treatment. Further dose‐range studies are necessary to determine adequate dosage and long‐term safety. Additionally, studies need to investigate the exact biochemical pathways impacted and look at the long‐term impact of ECD. Finally, research into ECD’s impact on other key metabolic tissues, such as the liver and muscle, would provide a more complete picture of its therapeutic potential.

## 4 Experimental Section

### 4.1 Human Bone Marrow Mesenchymal Stem Cell Culture

The immortalized human bone marrow mesenchymal stem cells (hMSCs) were obtained from the American Type Culture Collection (ATCC, USA). These are adult, adherent human cells derived from bone marrow. Donor sex and ethnicity are lot‐specific as reported by ATCC, and the cells were cryopreserved at passage two. As mentioned by the supplier, the hMSCs cell line used is a well‐characterized and authenticated human bone marrow‐derived mesenchymal stem cell line supplied by ATCC. This cell line has not been recorded as misidentified or contaminated by viral, fungal, bacterial, or mycoplasma sources, as guaranteed by ATCC. Authentication was guaranteed by ATCC quality control and paperwork provided at the time of purchase. The cells were cultured and maintained in Dulbecco’s Modified Eagle Medium (DMEM) supplemented with 10% fetal bovine serum (FBS) (Gibco‐Invitrogen, USA) at 37°C in a 5% CO_2_ incubator.

### 4.2 Adipocyte Differentiation

The experiments for in vitro cell culture, adipocyte differentiation, and maintenance were carried out according to the guidelines of the institutional research committee, King Saud University, Riyadh, KSA. Briefly, hMSC cells were plated at a density of 15,000 cells/cm2 as a monolayer and cultured with basal media. At 70% confluency, hMSC cells were induced for adipocyte differentiation using standard induction/maintenance media containing rosiglitazone, insulin, dexamethasone (DEX), and 1‐methyl‐3‐isobutyl xanthine (IBMX) (Sigma–Aldrich, St. Louis, MO, United States), as described before [34]. After 72 h, the induction media were replaced with maintenance media containing recombinant human insulin alone and incubated for 48 h [35]. Differentiation‐induced cells were further maintained by changing media every 3 days, according to the experimental design.

### 4.3 Epoxy Clerodane Diterpene Source and Isolation Procedure

The active compound, ECD (IUPAC Name: 5*R*, 10*R*)‐4*R*, 8*R*‐dihydroxy‐2*S*, 3*R*:15, 16‐diepoxycleroda‐13(16), 17, 12*S*:18,1*S*‐dilactone) was obtained and identified from the seeds of *Cassia tora* (*Caealpiniaceae*), originally stored in the Herbarium of the Faculty of Pharmacy of King Saud University (Index SY‐220 Voucher number 16 055). As previously described, 500 gm of the leaves of *Cassia tora* were collected, rinsed with distilled water, shade dried, powdered, and extracted by the cold percolation method [10]. The extract identification was performed by chromatography using 100–200 mesh Silica gel. Dissolving the extract in a chloroform /methanol mixture at a ratio of 19:1 resulted in a highly polar white compound, which was tested in Noller’s Test by mixing with tin and thionyl chloride to convert to pink, revealing that the compound is a terpenoid [36]. Repeated treatment and crystallization from methanol resulted in transparent crystals of ECD as previously noted [10].

### 4.4 Cytotoxicity Assay

The cytotoxicity of ECD (Sigma–Aldrich, St. Louis, MO, United States) was determined using a modified Methyl Thiazolium Tetrazolium (MTT) method [37]. Briefly, differentiated adipocytes were seeded at a density of 104 cells/well onto flat‐bottom 96‐well culture plates and treated with different doses of ECD (0, 0.5, 1, 2, 4, 8, 16, 32, 64, and 128 μM) for 24 and 48 h. ECD was dissolved in DMSO, and the final DMSO content in both the treatment and control groups did not exceed 0.1% (v/v). The negative control group was prepared with the same concentration of DMSO (vehicle control) but without ECD. Later, 10 µl of MTT solution (1 mg/ml) was added to each well and incubated in the dark for 4 h. The resulting violet crystals of formazan were solubilized using DMSO (10%). The absorption of solubilized purple colored solution was measured at *λ* = 570 nm in a BioTec Synergy multi‐well ELISA plate reader (Agilent Technologies, Inc., Santa Clara, CA, United States).

### 4.5 Dose Determination

The differentiated adipocytes (3rd day) were treated with three different concentrations (1, 2, 4, and 8 µM) of ECD (dissolved in DMSO) and maintained up to the tenth day. Further, the selection of an effective dose has been carried out according to the lipid accumulation inhibitory potential of ECD after 14 days [35]. To confirm the lipolytic effect, the differentiated adipocytes were treated with ECD on the third day and maintained until day 14, while the maintenance media were replaced every 3 days. In separate settings, the differentiated cells were treated with Orlistat (8 µM) or quercetin (8 µM), as reference drugs, as described above.

### 4.6 Oil Red O and Nile Red Staining

To analyze the accumulation of intracellular triglyceride, the treated cells were fixed in 4% paraformaldehyde at room temperature for 30 min. The fixed cell was stained with Oil Red O (1‐((4‐(Xylylazo)xylyl)azo)‐2‐naphthol) or Nile red solution (Sigma–Aldrich, St. Louis, MO, United States) for 30 min at room temperature; the unbound dye was washed with 70% ethanol and distilled water. Then, the morphology of lipid accumulation images of ECD‐treated cells was obtained with an inverted microscope [38]. The bound oil red O stain was extracted using 40% acetone to relatively quantify the accumulated lipid after gentle removal of unbound dye. A UV‐Spectrophotometer was used to measure the optical density of the extracted dye, which is relatively proportional to the lipid accumulation level compared to the untreated control.

### 4.7 Mitochondrial Membrane Potential (JC‐1 Staining) Assay

The untreated and ECD‐treated maturing adipocytes have been prepared in 24‐well culture plates. Subsequently, a 1:1 ratio of culture medium and JC‐1 staining solution was mixed and incubated in each well. After 20 min of incubation in the dark at 37°C, the dye was gently removed and washed twice with 100 µl of JC‐1 wash buffer (4°C). The fluorescence of each well has been observed under a fluorescence microscope, and images were recorded.

### 4.8 Analysis of Inflammatory and Browning Factors‐Related Gene Expression

Total RNA from different experimental groups has been collected, and the cDNA was prepared directly by the Fastlane Cell cDNA kit (QIAGEN, Germany), according to the manufacturer’s instructions. The genetic analysis of the targeted genes was performed by gene‐specific SYBR Green‐based QuantiTect Primer assays (QIAGEN, Germany) and the quantitative reverse transcription‐PCR system (Applied Biosystems 7500 Fast, Foster City, CA). These genes included UCP‐1, PRDM16, PPARγC1α, SREBP1c, C/EBPα, PPARγ, Adipo‐R1, LPL, and the reference gene β‐actin. The gene expression levels have been validated as previously defined [39]. The results have been expressed as Δ^Ct^ = Ct (ECD treated)‐Ct (untreated control). The value was used to plot the expression of inflammatory and browning‐related genes using the expression of 2^‐ΔΔCt^. Thus, the mRNA expression ranges were expressed as n‐fold differences relative to the reference control.

### 4.9 Determination of Inflammatory Mediators

To quantify the inflammatory mediators, such as TNF‐α, STAT‐6, MCP‐1, and LTB4R, in untreated and ECD‐treated cells, the high‐sensitivity ELISA kits (Quantikine, Minneapolis, MN, United States) were used according to the manufacturer’s instructions.

### 4.10 Statistical Analysis

All experiments were repeated in triplicate. The obtained results were expressed as mean ± standard deviation (SD). The data results were analyzed by the SPSS/11.5 software package using the one‐way analysis of variance (ANOVA) followed by Tukey’s test [40]. At the probability values (P), *p* < 0.05 and *p* ≤ 0.001, the results were considered significant.

## Author Contributions

**Sahar Abdulaziz AlSedairy**, **Maha H. Alhussain**, **Manal Abdulaziz Binobead**, and **Pandurangan Subash‐Babu**: concept and design, analysis and interpretation, data collection, and article writing. **Maha H. Alhussain**, **Pandurangan Subash‐Babu**, **Manal Abdulaziz Binobead**, and **Ali A. Alshatwi**: critical revision of the article, final approval of the article, statistical analysis. **Pandurangan Subash‐Babu** and **Ali A. Alshatwi**: overall responsibility. **Sahar Abdulaziz AlSedairy**: obtained funding.

## Funding

This study was supported by the Deanship of Scientific Research, King Saud University (ORF‐2026‐178).

## Conflicts of Interest

The authors declare no conflicts of interest.

## Data Availability

The datasets generated during and/or analyzed during the current study are available from the corresponding author on reasonable request.
